# Comparison of beneficial factors for corneal wound-healing of rat mesenchymal stem cells and corneal limbal stem cells on the xenogeneic acellular corneal matrix in vitro

**Published:** 2012-01-20

**Authors:** Jing Zhang, Chen Huang, Yun Feng, Ying Li, Wei Wang

**Affiliations:** 1 Department of Ophthalmology, Peking University Third Hospital, Beijing, China; 2 Medical Research Center of Peking University Third Hospital, Beijing, China

## Abstract

**Purpose:**

This experiment aims to investigate the potential ability of mesenchymal stem cells (MSCs) to produce beneficial factors for corneal recovery when the cells were seeded on a xenogeneic acellular corneal matrix (ACM) in vitro.

**Methods:**

MSCs and corneal limbal stem cells (LSCs) from the corneal limbus region of rats were isolated and cultured. Batch 1 from each type of cell was seeded on a Beagle cornea ACM at a density of 3×10^3^ cells/mm^2^ for 7 days. The proliferation activity of the cells was quantitatively determined at 1, 3, 5, and 7 days with a 3-(4,5-dimethylthiazol-2-yl)-2,5-diphenyltetrazolium bromide (MTT) assay. Keratin3/12 (CK3/12) growth factors, including vascular endothelial growth factor (VEGF), pigment epithelium-derived factor, epidermal growth factor, and transforming growth factor-beta1, integrin subunits such as α_5_β_1_, and α_6_β_1_, and elements of the extracellular matrix (ECM) such as fibronectin and laminin were investigated with immunofluorescence staining, reverse transcriptional polymerase chain reaction, and western blot assay, respectively.

**Results:**

The basic expression of growth factors in MSCs was much higher (n=6, p<0.05) than that in the LSCs, including VEGF, epidermal growth factor, and transforming growth factor-beta1. After being seeded on the ACM, those factors in MSCs expressed continuously at a high level, but the seeded corneal epithelium cells presented a downregulated trend in these factors. The expression of VEGF in seeded MSCs decreased, which was similar to the trend for the seeded LSCs (n=6, p<0.05). The expression of keratin3, a sign of mature epithelium cells, was also present in the MSCs after being seeded for 7 days. The expression of pigment epithelium-derived factor by the seeded and normal culture MSCs was equal, while the expression of this factor was not detected in either the seeded or the normal cultured LSCs. There were no significant differences between the integrin subunits (α5, α6, β1) and the extracellular matrix, including fibronectin and laminin, generated by normal cultured or seeded MSCs and LSCs.

**Conclusions:**

Under the ACM microenvironment, the MSCs presented beneficial factors for corneal recovery comparable to those presented by corneal LSCs. This indicates that MSCs, when combined with an ACM, may compose a competent corneal substitute for healing corneal wounds.

## Introduction

Today more than 10 million individuals worldwide suffer from corneal blindness [1] caused by ocular diseases and damage such as erosion due to viral or bacterial infections, chemical burns, neuroparalytic cornea, autoimmune diseases, and severe trauma [2]. Corneal transplantation using human donor tissue is one feasible treatment, but a severe shortage of cornea donors makes such transplantation increasingly difficult. In recent years, this shortage has been aggravated by the aging population (which increases demand) [3] and the increased use of corrective laser surgery (which decreases the supply of tissue) [4].

Bioengineered corneal substitutes comprise a novel sector for research in the field of reconstructing ocular surfaces. The substitute, based on using special cells and a biomaterial scaffold, is already available for experimental application [5]. The reconstructed corneal tissue represents a fascinating step toward considering transplantations as “replacement parts,” or in enhancing wound healing in vitro [6-8]. Therefore, the scaffold and the starting cells should be chosen carefully.

As described in our earlier report [9], an acellular corneal matrix (ACM) has been used as a scaffold; tissue produced from the ACM exhibited physical and mechanical characteristics very similar to normal cornea tissue, including its strength, rate of expansion, water content, and light transparency. Furthermore, the ACM provides a suitable microenvironment for three kinds of corneal cells to grow. Isolating the appropriate type of cell to seed seems to be extremely important, since the quality of the reconstructed tissue will vary greatly according to the quality of the starting cellular material. The ideal source for cells used in tissue reconstruction would provide cells with extensive proliferation potential (self-renewal capacity) and the ability to differentiate appropriately (able to produce differentiated progeny).

We have already learned that limbal stem cells (LSCs) are a major renewable source of epithelium cells from the cornea. LSCs have a great potential flourish in explant cultures after being seeded on the ACM [10]. Because of the exiguous nature of autograft material, however, harvesting LSCs may not be suitable for binoculus sufferers who are unwell, because that strategy risks damaging the comparatively healthy eye. The key to resolving this problem is to find cells that can be collected easily in sufficient quantity.

Mesenchymal stem cells (MSCs) have attracted attention as a better treatment option with little or no immunogenic potential. MSCs from adult human bone marrow are capable of differentiating into various cell lineages, e.g., cardiac muscle cells in vivo [11], neural cells in vitro [12], and limbus-like stem cells in vivo [13,14]. Furthermore, MSCs can be used as feeder cells to provide nutrient substance for adult and stem cells [15].

We hypothesized that in addition to transdifferentiating into epithelial-like cells MSCs seeded on the ACM could provide nutrient substance that performed as beneficial factors in the process of healing corneal wounds. We examined the hypothesis by comparing the growth factors, the integrins, and the extracellular matrix of the seeded LSC and MSC cells at the mRNA (mRNA) and the protein levels, respectively.

## Methods

### Animals

Male inbred Sprague Dawley (SD) rats (Vital River Laboratories, Beijing, China) aged 4 to 6 weeks were used for the cell culture. All the rats were treated in accordance with the Statement on the Use of Animals in Ophthalmic and Vision Research of the Association for Research in Vision and Ophthalmology (ARVO).

Rats were sacrificed with an overdose of 30% ethyl aminoformate (0.03 ml/kg); the eyeballs and the femurs were collected immediately under sterile conditions.

### Acellular corneal matrix

Fresh corneas from dogs (breed: Beagles) were obtained from the Experimental Animal Center of Peking University Third Hospital, Beijing, China.

First, the corneal epithelium and endothelium were removed using Dispase II (1.2 U/ml, Roche Applied Science, Penzbeg, Germany) at 4 °C for 16 h. The stroma was then trimmed to a 10 mm diameter section. To remove the hereditary material, the stromal discs were soaked in 1 mM Tris-HCl for 12 h, treated with a 1% Triton X-100 solution (Sigma-Aldrich, St. Louis, MO) at 4 °C for 12 h, digested with 0.25% trypsin-EDTA (Invitrogen, Carlsbad, CA) at 37 °C for 30 min, and treated with DNase (Sigma) and RNase (Sigma) at 4 °C for 16 h. Between the treatment steps, the discs were washed twice with 5 mM Tris-HCl for 10 min. Finally, the scaffold materials were freeze-dried at −20 °C for 8 h, 0 °C for 8 h, and 20 °C for 4 h and then sterilized with gamma irradiation.

The histological characteristics and nucleus of the ACM were detected with hematoxylin and eosin (HE) stain and 4’,6-diamidino-2-phenylindole (DAPI) stain [16-19].

### Isolation and culture of corneal limbal stem cells

Before cell dissection, the rat corneal tissues were rinsed three times with PBS (with 100 IU/ml penicillin and 25 μg/ml gentamicin; Sigma). After excess sclera, the iris, the corneal endothelium, conjunctiva, and Tenon’s capsule were carefully removed, the limbal rings were incubated at 37 °C for 1 h with 1.2 U/ml Dispase II.

The limbal epithelial sheets were separated from the residual corneal stroma under a dissecting microscope using two fine forceps with gentle horizontal movements, and further digested with 0.25% Trypsin/0.02% EDTA (Sigma) at 37 °C for 5 min to isolate single cells. We used a 1:1 mixture of Dulbecco’s minimal essential medium and Ham’s F12 medium (DMEM/F12; Invitrogen), as a growth medium, supplemented with 15% fetal bovine serum (FBS; Invitrogen), 100 U/ml penicillin, 25 μg/ml gentamicin, 10 ng/ml epithelial growth factor (EGF; Sigma), 5 μg/ml insulin (Sigma), 10 nM cholera toxin (Sigma) and 5 μg/ml transferrin (Sigma). All cultures were maintained under standard conditions at 37 °C in a humidified atmosphere containing 5% CO_2_. The growth medium was replaced every two days.

### Isolation and culture of bone marrow–derived mesenchymal stem cells

The ends of the diverticula part of the rat femurs were cut, and the bone marrow cavity was washed four to five times with Dulbecco’s minimal essential medium-Low Glucose (DMEM-LG; Invitrogen) supplemented with 10% FBS.

The fluid was resuspended in fresh complete medium at a cell density of 10^6^–10^7^/ml and then placed directly onto plastic 100 mm Petri dishes that were maintained in a humidified atmosphere of 5% CO_2_ at 37 °C. The medium was changed every two days, and the proliferation and morphological characteristics of the cells were observed daily under an inverted microscope (Nikon TE2000-S; Nikon Corporation, Tokyo, Japan).

### In vitro differentiation of mesenchymal stem cells

Batch 1 of the MSCs were seeded at a density of 5×10^3^ cells/well (six-well plate) and incubated overnight. Osteogenic differentiation was induced using an osteogenesis differentiation medium composed of DMEM supplemented with 0.1 μmol/l dexamethasone (Sigma), 50 μg/ml L-ascorbic acid (Sigma), 10 mmol/l β-glycerophosphate (Sigma), and 10% FBS. The medium was changed twice a week over a period of 21 days.

To visualize osteogenic differentiation, cells were detected with von Kossa staining. Adipogenic differentiation was induced using DMEM supplemented with 1 μmol/l dexamethasone, 0.5 mmol/l IBMX (Sigma), 10 μg/ml insulin (Sigma), 100 μg/ml indomethacin (Sigma), and 10% FBS. The induction medium was changed every three to four days, and cells were cultured for at least 14 days.

To demonstrate the presence of adipocyte-like cells, cells were fixed with 10% neutral-buffered 4% formalin for 10 min, and then cytoplasmic inclusions of neutral lipids were stained with oil-red O (3 mg oil red/ml 60% isopropanol) for 15 min.

### Limbal stem cells and mesenchymal stem cells seeded on the acellular corneal matrix

The sterilized ACM was split into small round pieces 10 mm in diameter and 2 mm thick. They were soaked in the DMEM/F12 medium supplemented with 10% FBS for 48 h before cell seeding. Batch 1 from each of the cultured MSCs and LSCs was harvested to inoculate on the ACM surface at a density of 3×10^3^/mm^2^. The two types of seeded cells were described as MSCs-A and LSCs-A.

After being seeded, the ACM was cultured in the 5% CO_2_, 37 °C incubator for an initial 1 h without adding more culture medium. After 1 h, sufficient DMEM/F12 containing 10% FBS was added to submerge the ACM. The culture media was very gently changed every two days. The morphological characteristics of the cells were observed daily under an inverted phase contrast microscope.

The proliferation activity of the seeded cells and the normal cultured cells were quantitatively determined at 1, 3, 5, and 7 days with a 3-(4,5-dimethylthiazol-2-yl)-2,5-diphenyltetrazolium bromide assay (MTT); the normal cultured cells were detected as the normal control. The optical density (OD) value of absorbance at 490 nm was measured with a microplate reader (InTec Reader 2006; Intec Products, Inc., Xiamen, China). Differences in the OD value between the two types of cells were analyzed statistically using a one-way ANOVA. Statistical significance was set at p<0.05.

### Scanning electron microscopy

LSCs and MSCs cultured on the ACM for seven days were fixed with 2% glutaraldehyde (Sigma) in 0.1 M PBS (pH 7.2) at 37 °C for 1 h, washed three times in buffer for 10 min, fixed in 1% osmium tetroxide (Sigma) for 1 h at 37 °C, and dehydrated through a graded series of ethanol. The fixed seeded LSC and MSC ACM specimens were critical point dried (HCP-2; Hitachi, Ibaraki, Japan), coated with platinum using an Ion Coater (IB-5; EIKO, Ibaraki, Japan), and observed on a scanning electron microscope (SEM) (Hitachi S-800; Hitachi) at 2000× magnification.

### Immunofluorescence

#### Cells characteristics detected

Immunofluorescence (IF) staining was used to detect CD29 (1:100; Chemicon, Temecula, CA), CD45, and CD34 (1:100; Santa Cruz Biotechnology, Inc., Santa Cruz, CA) for the cultured MSCs. In addition, P63, ABCG2, and CK3/12 (1:100; Abcam, San Francisco, CA) were checked for the cultured LSCs. The LSCs and MSCs from Batch 1 were fixed in ice-cold methanol on a culture plate for 10 min and washed in PBS. After being blocked with 2% BSA in PBS, the primary antibodies were applied overnight in a moist chamber set at 4 °C and then rinsed with PBS three times for 5 min. They were then incubated with the 1:200 diluted tetraethyl rhodamine isothiocyanate (TRITC)–conjugated-goat and antimouse immunoglobulin G secondary antibodies (Santa Cruz Biotechnology, Inc.) for 45 min in a dark incubation chamber at 37 °C.

After the two kinds of cells were washed in PBS, DAPI (1:1000; Invitrogen) was used for nuclear staining. All specimens were examined under a fluorescence microscope. The negative controls were prepared by incubation with the secondary antibody alone.

#### Characteristics of the cell–acellular corneal matrix scaffold

The cell–ACM scaffold specimens were freshly frozen in an optimal cutting temperature compound (OCT; Sakura Finetek, Torrance, CA) and then sliced with a cryostat. Section tissues were air dried and blocked with 2% BSA in PBS.

Cells seeded on the ACM were detected with primary antigens of Keratin3/12 (CK3/12, 1:100; Abcam), growth factor, integrin, and the ECM. The ECM contained vascular endothelial growth factor (VEGF, 1:100; Abcam), pigment epithelium–derived factor (PEDF, 1:100; Abcam), epidermal growth factor (EGF, 1:50; Abcam), transforming growth factor-beta1 (TGF-β1, 1:100; Abcam), integrin subunits α5β1 and α6β1 (1:100; Chemicon), fibronectin (FN, 1:200; Abcam), and laminin (LM, 2:100; Abcam). The process of IF staining was performed as described above. Fluorescein isothiocyanate (FITC)–conjugated-goat and antimouse immunoglobulin G were used as the secondary antibodies (1:200; Santa Cruz Biotechnology, Inc.), and propidium iodide (1:2000; Invitrogen) was used for nuclear staining.

All section tissues were observed on a confocal laser microscope (LSM 510; Zeiss, Oberkochen, Germany). The two Petri dish–cultured types of cells were stained in parallel as controls.

### Total RNA isolation and reverse transcriptional polymerase chain reaction

Total RNA was extracted from each sample (corneal epithelium cells, MSCs, MSCs-A, and LSCs-A) with TRIzol (Invitrogen). The RNAs were quantified by measuring absorption at 260 nm. RT–PCR was performed using the RevertAid^TM^ First Strand cDNA Synthesis Kit (Fermentas, Ontario, Canada). The first-strand cDNA was synthesized after incubation at 42 °C for 60 min. PCR conditions were 95 °C for 15 min followed by 35 cycles of 94 °C for 5 min, 94 °C for 40 s, the melting temperature (Tm) of each gene for 45 s, 72 °C for 1 min 2 s, and, finally, 72 °C for 10 min.

Table 1 presents the gene sequences of the primers for vascular endothelial growth factor (*VEGF*), pigment epithelium-derived factor (*PEDF*), epidermal growth factor (*EGF*), transforming growth factor-Subunit β1 (*TGF-β1*), integrin subunit α5β1 and α6β1, fibronectin (*FN*), and laminin (*LN*), as well as the housekeeping gene used as an internal control. We chose glyceraldehyde 3-phosphate dehydrogenase (*GADPH*) as a reference. Each sample was repeated three times.

**Table 1:** The sequences of the primers for each factor.

**Gene**	**Primer**	**Product length (bp)**
VEGF	Forward 5′-GAGTATATCTTCAAGCCGTCCTGT-3′	230
	Reverse 5′-ATCTGCATAGTGACGTTGCTCTC-3′	
*PEDF*	Forward 5′- CTGGCAACCCTCGCATAG −3′	201
	Reverse 5′- TGTCCTCGTCCAAGTGAAA −3′	
*EGF*	Forward 5′-ATGTCTGCCAATGCTCAGAAGG-3′	617
	Reverse 5′-TAGGACCACAAACCAAGGTTGGG-3′	
*TGFβ1*	Forward 5′-CGAGGTGACCTGGGCACCATCCATGAC-3′	404
	Reverse 5′-CTGCTCCACCTTGGGCTTGCGACCCAC-3	
Integrinα5	Forward 5′-TCTGCCTCAATGCTGG-3′	248
	Reverse 5′-GTTGAGATCTATGTGAATCG-3′	
Integrinα6	Forward 5′-TTGGTAGCTACTGTGAGGTCGAAAC-3′	395
	Reverse 5′-ACCATTGAATCCGACAGCCAC-3′	
Integrinß1	Forward 5′-ACACGTCTCTCTCTGTCG-3′	158
	Reverse 5′-CAGTTGTTACGGCACTCT-3′	
Fibronectin	Forward 5′-CCGTGGGCAACTCTGTC-3′	438
	Reverse 5′-TGCGGCAGACAGTTGTC-3′	
Cytokeratin	Forward 5′-GCTGAGAGTTTGTTTACCGT-3′	356
	Reverse 5′-AGTGATTCATCCTTTCCAAT-3′	
Laminin	Forward 5′-ACGGCAAGGTGTTGTGCGAT-3′	294
	Reverse 5′-GCTGGGGAGCAAACTTTCCT-3′	
*GADPH*	Forward 5′-TATCGGACGCCTGGTTAC-3′	407
	Reverse 5′-TGCTGACAATCTTGAGGGA-3′	

The amplified products were separated using 1% agarose gel electrophoresis stained with Gold View^TM^ (SBS Genetech, Beijing, China). Densitometry analysis was performed on scanned PCR-production pictures and quantified according to pixel intensity (Quantity One 4.1.0 software; Bio-Rad Laboratories, Hercules,CA). The densitometry values obtained for each factor were subsequently normalized to the *GADPH* level in the same blot.

### Protein isolation and western blot analysis

The Batch 2 cells of each sample were harvested for western blot analysis. The cells were sonicated for 15 s intervals at 100 W. Cell debris was cleared from the lysates by centrifugation at 200× g for 15 min at 4 °C. The lysates were separated into soluble and particulate fractions using centrifugation at 29,000× g for 30 min at 4 °C. The pellets were resuspended in modified radio immunoprecipitation assay buffer (50 mM Tris-HCI, pH 7.4), 1% Nonidet p-40, 0.25% sodium deoxycholate, 150 mM sodium chloride, 1 mM EDTA, 1 mM phenylmethanesulfonyl fluoride, 5 μg/ml aprotinin, 5 μg/ml leupeptin, 1 μg/ml pepstatin, 1 mM Na_3_VO_4_, and 1 mM sodium fluoride.

Protein concentrations were determined with the bicinchoninic acid protein assay reagent kit (Pierce Biotechnology, Rockford, IL). The supernatant and particulate fractions were stored at −80 °C. Sodium dodecyl sulfate–PAGE was performed on Bis-Tris 4%−12% gels (NuPAGE; Novex, San Diego, CA) with 2-N-morpholino-ethanesulfonic acid running buffer (Invitrogen).

Proteins were detected on the immunoblots with chemiluminescence (SuperSignal Femto West Chemiluminescent Substrate Kit; Pierce) according to the manufacturer’s directions. Blots were developed on an autoradiograph film (CL-Xposure; Pierce and GBX developer and fixer solutions, Eastman Kodak, Rochester, NY). Blots were reused after the antibodies, and substrates were removed with the western blot stripping buffer (Restore; Pierce) according to the manufacturer’s directions.

Antibodies used in these studies included anti-PEDF, anti-EGF, anti-TGFβ1, anti-VEGF, and anti-CK3. The β-actin protein was chosen as a reference. Each sample was repeated three times. Densitometry analysis was performed on scanned autoradiographic films and quantified according to pixel intensity (Quantity One 4.1.0 software; Bio-Rad Laboratories). The densitometry values obtained for each factor were subsequently normalized to the β-actin level in the same blot.

### Statistics

Data were presented as mean±standard error of mean (SEM). Two-sided paired or non-paired Student *t* tests were used to compare the mean values of two sets of data. Statistical analyses were performed using SPSS for Windows, Version 11.0 (SPSS Inc., Chicago, IL). Differences were considered significant at p<0.05.

## Results

### Histological characteristics of the acellular corneal matrix

**Figure 1:**
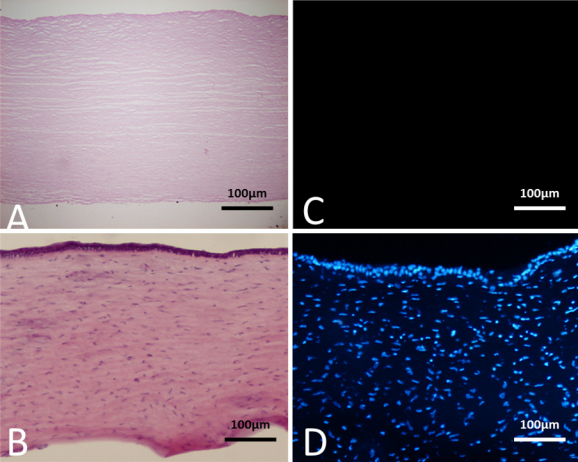
Histological characteristic of the acellular corneal matrix (ACM). Sections of the ACM stained with HE and DAPI presented a regular structure and no visible cell (**A**; 20×) or nuclear material (**C**; 20x) when compared with the normal cornea, (**B**; 20×) and (**D**; 20×), respectively.

The ACM produced from the Beagle cornea has no visible cell or nuclear material with HE staining and DAPI staining, keeping only the regular scaffold similar to that found in the normal Beagle cornea (Figure 1A-D).

### Culture and identification of corneal limbal stem cell cells

**Figure 2:**
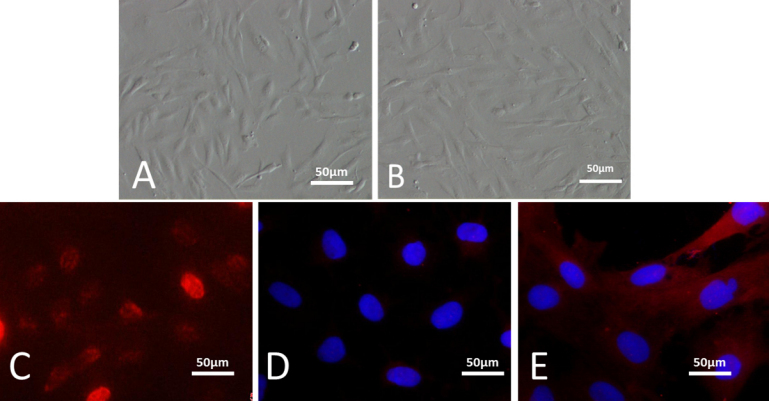
Limbal stem cells cultured in vitro. Inverted phase contrast microscopy showed the morphology of limbal stem cells. Cells appeared to be compact, uniform, and cobblestone-pavement in shape (**A**; 20×), with 70% confluence at day 7 (**B**; 20×). The overwhelming majority of cells were positive for p63 (**C**; 20×) and ABCG2 (red; **E**; 20×) indicated the presence of limbal epithelial progenitor cells, only a few of which were keratinK3/12 positive (red merged with DAPI; **D**; 20×).

As early as 12 h after seeding, cells adhered on the Petri dish. The morphology of epithelial cells from the limbal region in the primary culture appeared to be compact, uniform, and cobblestone-pavement in shape (Figure 2A).

By day 7, the cells were at 70% confluence (Figure 2B). IF staining revealed that a small portion of the limbal epithelial cells were positive for keratinK3/12 (Figure 2D), and the overwhelming majority of these cells were positive for p63 (Figure 2 C) and ABCG2 (Figure 2E). The p63 and the ABCG2 indicated a predifferentiation status, which suggests the high proliferation of cultured cells.

### Culture and identification of mesenchymal stem cells

**Figure 3:**
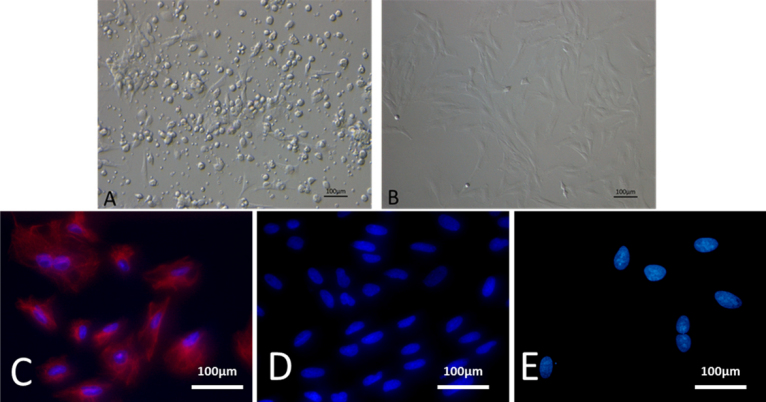
Mesenchymal stem cells cultured in vitro. Mesenchymal stem cells cultured on day 3 (**A**; 10×) and day 5 (**B**; 10×). Immunofluorescent staining of CD29 was positive (red merged with DAPI; **C**; 20×); CD34 (**D**; 20×) and CD45 (**E**; 20×) were negative.

Three days after culture, the MSCs began to stretch (Figure 3A). The non-adherent cells were removed by changing the culture media. The spindle-like MSCs were distributed widely. There were larger clones on day 5; the cells grew rapidly and became fibroblast-like with rich cytoplasm and a large nucleus (Figure 3B). IF staining showed that MSCs expressed the mesenchymal cell marker CD29 (Figure 3C) but did not express hematopoietic stem cell markers CD34 (Figure 3D) or CD45 (Figure 3E).

### Differentiation of mesenchymal stem cells in vitro

**Figure 4:**
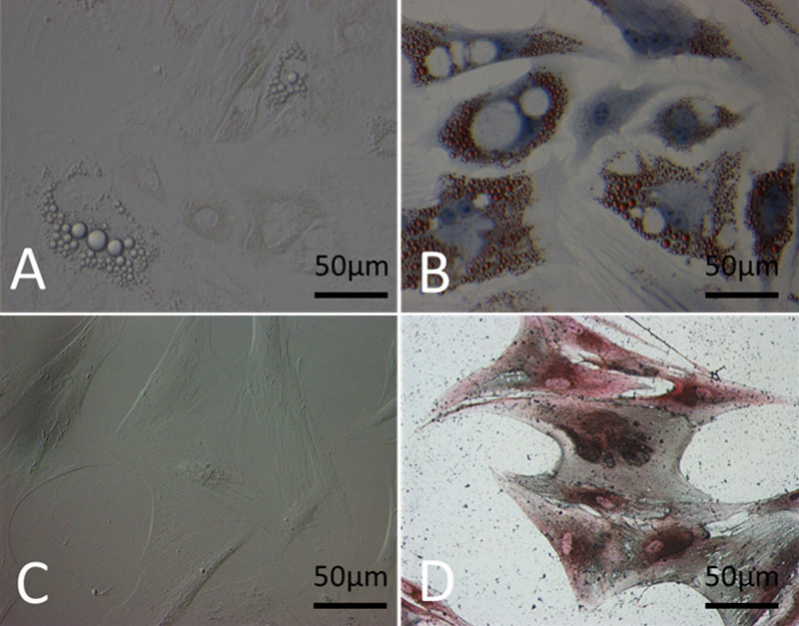
Multi-differentiated MSCs. Lipid droplets appear in the cytoplasm at the fourth day (**A**; 20×), and large numbers of lipid droplets (red) in MSCs are shown with Oil-red O staining after adipogenesis induction (**B**; 20×). Under osteogenic culture conditions, MSCs become flat and wide (**C**; 20×) and calcium nodules (black) can be detected in the cytoplasm with von Kossa staining (**D**; 20×).

The plastic-adherent MSCs were multipotent and capable of differentiation into adipocytes and osteoblasts when they were cultured in adipogenic and osteogenic induction medium, respectively [20]. To demonstrate differentiation into these cell types, oil-red O and von Kossa stainings were performed. The lipid droplet can be seen in the cytoplasm as early as the fourth day (Figure 4A). Oil-red O staining showed large numbers of lipid droplets at day 14, indicating induction of adipogenesis (Figure 4B). Under osteoblast induction medium, the MSCs were flat and wide (Figure 4C). The osteogenesis was confirmed with von Kossa staining to detect the formation of calcium nodules in the cytoplasm (Figure 4D).

### Morphology of cells after been seeded on the acellular corneal matrix

**Figure 5:**
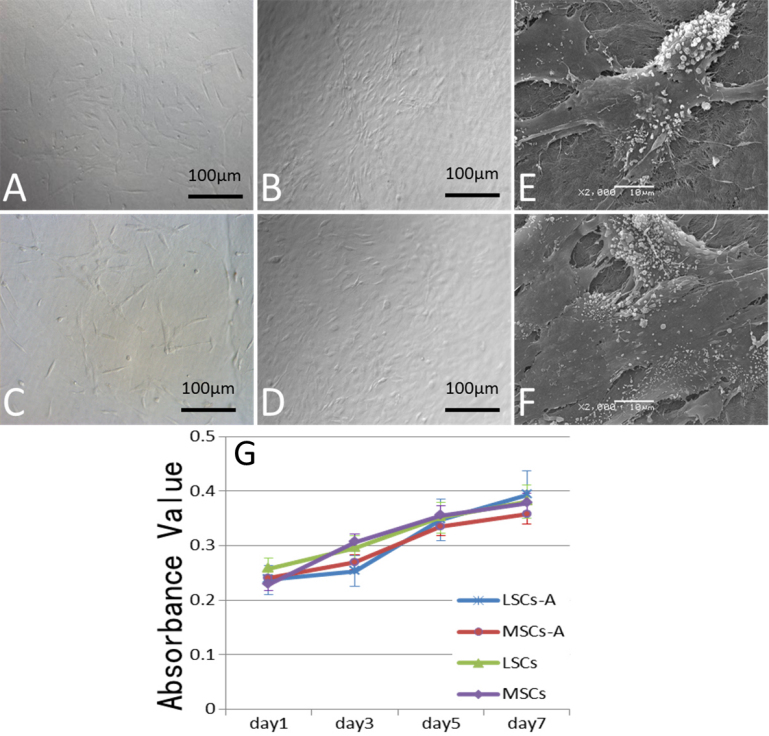
Morphology of the seeded cells. Mesenchymal stem cells (MSCs; p=1) adhered to the acellular corneal matrix (ACM) after 12 h (**A**; 10×), and after 7 days, the seeded MSCs showed a ceroid appearance (**B**; 10×). A scanning electron microscopic (SEM) showed that the MSCs adhered to the ACM tightly, with masses of microvilli on their apical surface (**E**; 2,000×). Corneal limbal stem cells (LSCs; p=1) adhered on the ACM after 12 h (**C**; 10×), and after 7 days (**D**; 10×). SEM showed a flat, squamous, polygonal appearance (**F**; 2,000×). Under the seeded microenvironment, MSCs and LSCs presented similar proliferation ability with cells under the normal culture condition. There were no significant differences in the proliferation of the seeded MSCs and corneal LSCs as determined with MTT (**G**).

As early as 12 h after seeding, LSCs and MSCs adhered to the surface of the ACM. Under the culture condition of DMEM/F12 (1:1) supplemented with 10% FBS, the MSCs (p=1) presented primarily a fibroblast-like morphology (Figure 5A). The seeded cells were at 80% confluence by day 7, and the appearance of the seeded MSCs became ceroid (Figure 5B). This epithelium-like shape indicated that the seeded MSCs might have transdifferentiated into epithelium cells.

The scanning-electron microscopy image indicated that the seeded MSCs adhered tightly on the ACM, with masses of microvilli on their apical surface (Figure 5E). The corneal LSCs (p=1) showed a cobblestone-pavement morphology (Figure 5C), which also reached 80% confluence at day 7 (Figure 5D). SEM images showed a flat, squamous, polygonal appearance (Figure 5F). Under the seeded microenvironment, MSCs and LSCs presented similar proliferation ability with cells under the normal culture condition. There were no significant differences in the proliferation of the seeded MSCs and corneal LSCs as determined with a 3-(4,5-dimethylthiazol-2-yl)-2,5-diphenyltetrazolium bromide assay (n=3, three samples from ADM sheet with two types of seeded cells for the seeded groups; three samples from normal cultured cells in 24-well board for the normal cultured groups, p>0.05; Figure 5G).

LSCs and MSCs showed a high proliferation rate when seeded on the ACM, indicating that the matrix can provide a suitable environment for these two types of cells. Furthermore, the seeded MSCs presented epithelium-like shapes. This result indicated that under the ACM microenvironment, the seeded MSCs potentially might be able to transdifferentiate into corneal LSCs.

### Immunofluorescence stain qualitatively detected expression of keratin, growth factors, extracellular matrix, and integrins

**Figure 6:**
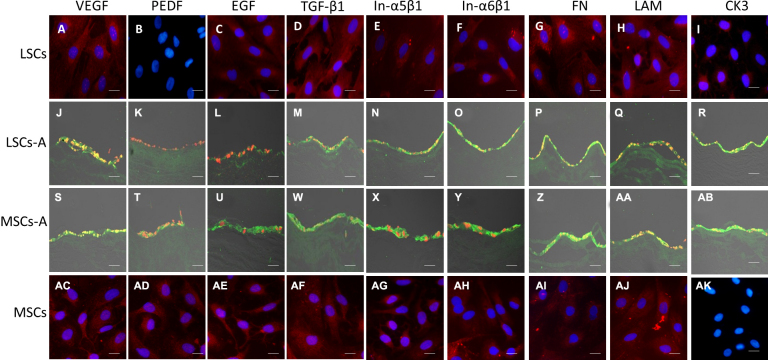
Expression of keratin, growth factors, the ECM, and integrins was detected qualitatively with immunofluorescence stain. The positive signal for normal cultured cells was red; the nuclei are stained with DAPI (blue). For seeded cells, the positive signal was green, and nuclei are stained with PI (red).

IF staining confirmed that when compared with the normal cultured MSCs and LSCs, the seeded MSCs (MSCs-A)and seeded corneal limbal stem cells (LSCs-A) uniformly expressed growth factors, including VEGF (Figure 6A,J,S,AC), integrins including subunits α5β1 (Figure 6E,N,X,AG) and α6β1 (Figure 6F,O,Y,AH), and the extracellular matrix (ECM) including fibronectin (Figure 6G,P,Z,AI) and laminin (Figure 6H,P,AA,AJ).

These results demonstrate that after being cultured on the ACM microenvironments MSCs and the LSCs still kept the ability to perform VEGF, ECM, and integrins synthesis. The EGF was positive in normal cultured MSCs and corneal LSCs (Figure 6C,AE), and was still positive in seeded MSCs (Figure 6AE). However, it become partly positive in the seeded corneal LSCs (Figure 6L).

The TGF-β1 has the same trend as the EGF (Figure 6D,M,W,AF). The PEDF in normal cultured and seeded MSCs was positive (Figure 6T,AD); however, the normal cultured and seeded corneal limbal stem cells were negative (Figure 6B,K).

The mature corneal epithelium marker keratin3/12 was positive in the normal cultured corneal LSCs (Figure 6I), but negative for the MSCs (Figure 6AK); after being seeded on the ACM, this marker became positive (Figure 6AB), which was similar to the seeded corneal epithelium cells (Figure 6R). The changed expression of CK3 in MSCs indicated that the seeded cells had the ability to transdifferentiate into corneal epithelium cells.

### Reverse transcriptional polymerase chain reaction assay

**Figure 7:**
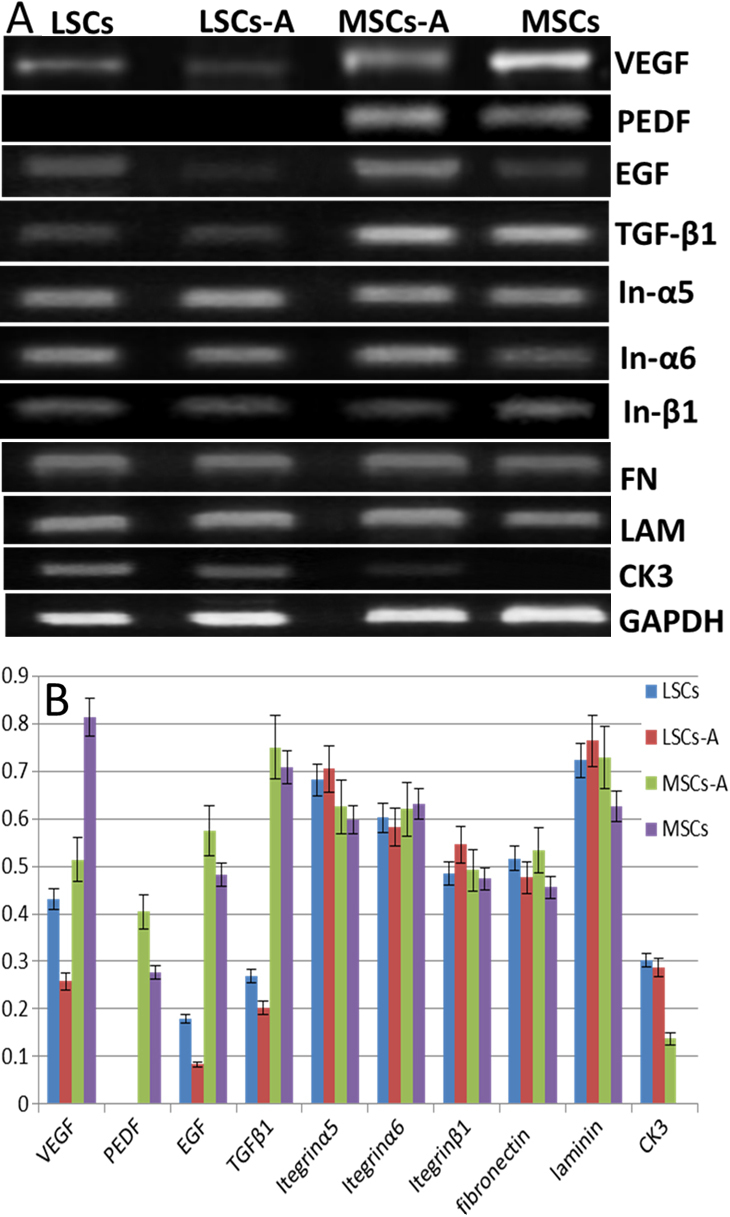
RT–PCR analysis. The keratin, growth factors, integrins, and the ECM at the mRNA level (**A**). Different factors/*GAPDH* ratio (**B**). Values are expressed as mean±SEM (n=6, six samples from ADM sheet with two types of seeded cells for the seeded groups; six samples from normal cultured cells in 24-well board for the normal cultured groups, p<0.05). Abbreviations: LSCs represents limbal stem cells; MSCs represents bone marrow deserved mesenchymal stem cells; LSCs-A represents LSCs seeded on the ACM; MSCs-A represents MSCs seeded on the ACM.

The gene expression of keratin, growth factors, ECM, and integrins at the mRNA level was assayed quantitatively with reverse transcriptional polymerase chain reaction (RT–PCR) (Figure 7A,B). According to the statistical results from comparing pixel intensity, the basic expression of growth factors in MSCs was much higher (n=6, p<0.05) than that in the corneal epithelium cells, which included *VEGF*, *EGF*, and *TGF-β1*.

After being seeded on the ACM, those factors expressed at a high level continuously in MSCs, but the expression in seeded LSCs presented a downregulated trend. The differences between the two types of seeded cells were significant (n=6, p<0.05). Interestingly, the expression of *VEGF* in seeded MSCs decreased, which was similar to the trend for seeded LSCs (n=6, p<0.05).

The expression of keratin3, a sign of mature epithelium cells, was also present in the MSCs after being seeded for seven days. The expression of *PEDF* in the seeded and normal culture MSCs was equal, which could not be detected in either the seeded or normal cultured corneal LSCs.

The mRNA of integrin subunits α5, α6, and β1 and of the ECM, including fibronectin and laminin, have no significant difference between the two types of normal cultured cells, or between the seeded cells (n=6, p<0.05).

### The diversity of the growth factor and keratin determined with western blot

**Figure 8:**
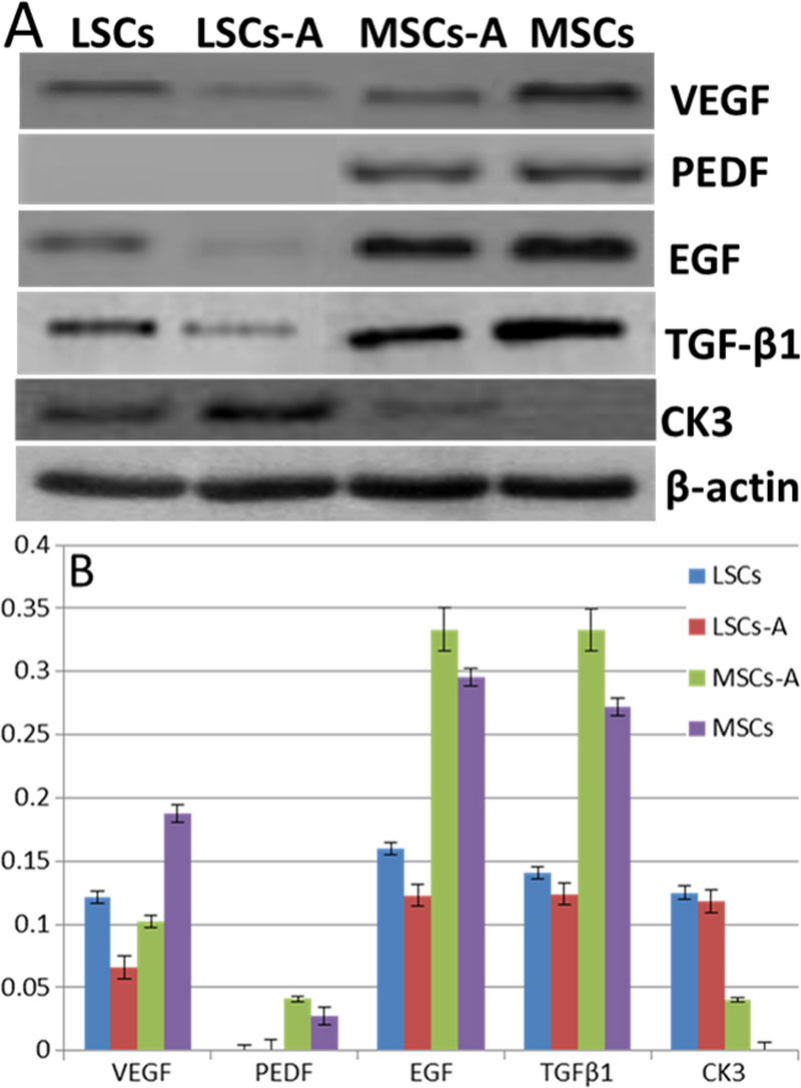
Western blot analysis. The diversity of the growth factor and keratin determined at the protein level (**A**). Ratio of different factors to β-actin (**B**). Values are expressed as mean±SEM (n=6, six samples from ADM sheet with two types of seeded cells for the seeded groups; six samples from normal cultured cells in 24-well board for the normal cultured groups, p<0.05). Abbreviations: LSCs represents limbal stem cells; MSCs represents bone marrow deserved mesenchymal stem cells; LSCs-A represents LSCs seeded on the ACM; MSCs-A represents MSCs seeded on the ACM.

According to the RT–PCR results, four growth factors and keratin3 were different in the comparison of the basic cultured cells and the ACM-seeded cells. To see if the differences existed at the protein level, a western blot was conducted. The results showed that the variances of those proteins were consistent with the mRNA (Figure 8A,B).

## Discussion

Clearly, the most competent cells to use for corneal reconstruction are the autologous corneal limbus stem cells. However, binoculus sufferers need an alternative source for cells.

The stromal cell population in bone marrow contains multipotent MSCs capable of differentiating into several non-hematopoietic phenotypes, including endothelial cells [21,22], chondrocytes [23], osteoblasts [24], adipocytes, brain cells [25], fibroblasts, keratinocytes [26], and myogenic cells. Because of their extraordinary plasticity and their ability to secrete restorative factors that promote tissue repair, MSCs have been tested as cellular therapy in preclinical models [27,28]. Consequently, MSCs may also offer better prospects in corneal reconstruction.

According to our earlier report and many other research studies [29], the xenogeneic ACM presented to be an ideal scaffold for cornea reconstruction has good biocompatibility, high optical clarity, toughness to withstand surgical procedures, and non-immunogenicity properties.

After finishing the preparation of two key elements of the corneal substitution, we tested whether the ACM-seeded MSCs could present characteristics similar to the seeded corneal epithelium cells for healing corneal wounds.

Corneal wound-healing is a complex, multistage process. For corneal wounds to heal optimally, a cascade of multicellular interactions and tissue remodeling must proceed in an orderly fashion [30]. This process can be enhanced by several tissue-repair growth factors secreted by tissue-borne cells and those recruited to the wound immediately after tissue injury. In fact, a deficiency in producing growth factors or a lack of such factors [31] available at the wound are major causes of compromised wound-healing in some diseases [32]. Studies have also revealed that MSCs can even migrate to the wound area and produce growth factors and cytokines after reaching the wounded milieu. The therapeutic efficacy of MSCs as a corneal substitute might be related to production of growth factors by these cells.

Our IF staining revealed that MSCs continued to express growth factors, including VEGF, EGF, PEDF, and TGF-β1, after being seeded on the ACM, whereas EGF and TGF-β1 were part positive in the seeded corneal LSCs. The assays of EGF and TGF-β1 at the mRNA and protein levels are in accordance with the IF, and the differences between the two seeded cells are significant. Since these two factors play important roles in the proliferation and migration of corneal epithelium cells in vivo and in vitro [33-35], this difference indicated that the ACM-seeded MSCs are superior to the seeded corneal LSCs in improving the proliferation and migration of cells.

The difference is also extremely meaningful for clinical use, where a persistent defect in epithelial cells will finally lead to corneal neovascularization [36,37]. The MSCs primarily expressed VEGF at a high level, but VEGF became downregulated after the MSCs were seeded on the ACM. There was no significant difference between the seeded MSCs and LSCs, which suggested the ACM-seeded MSCs carried with them no additional risk of inducing corneal neovascularization. Furthermore, the ability to synthesize PEFD may also keep the seeded MSCs away from neovascularization.

Healing corneal wounds obviously requires cells to migrate, proliferate, and adhere. Cell adhesion and migration depend in turn on synthesizing and assembling the ECM. The ECM is a complex, cross-linked structure made up of various proteins, such as FN, LM, collagen, and vitronectin, as well as polysaccharides. Among all the components from the ECM surrounding corneal cells, FN has been recognized as the prototypical cell attachment protein [38]. FN and LM are adhesion proteins identified as potential wound-healing agents because of their cell-attachment, migration, differentiation, and orientation properties [39,40]. Therefore, to propose using MSCs to improve healing corneal wounds, MSCs’ ECM expression must be very similar to that of keratocytes. In our study, the ECM in the ACM-seeded MSCs, including FN and LM, are comparable to the ACM-seeded corneal LSCs for mRNA and protein. This suggests that the seeded MSCs can provide a suitable environment for cells to regenerate and adhere properly.

Integrins are now clearly recognized as the main class of cell adhesion receptors for the various components of the ECM. Integrins can promote either cell-ECM or cell-cell interactions [41]. Researchers postulated that the increase in FN and LM expression in corneal wound-healing would be coordinated with the expression of their corresponding integrin receptors, α5β1 (FN) [42] and α6β1 (LM) [43]. Therefore, if one wished to find substitutable cells that are likely to share the many adhesive and migratory characteristics of the cells in a normal cornea, their corresponding patterns of integrin expression must be very similar. Our study shows that there was no significant difference in the synthesis of integrins between the LSCs and MSCs at the mRNA and protein levels, which also indicated that the seeded MSCs were a good alternative source of cell to use for healing corneal wounds.

In summary, this study revealed the characteristics of MSCs after they were seeded on the surface of the ACM. Compared with corneal LSCs, MSCs’ high level of expressing growth factors, the comparability of their abilities to integrins, and their synthesis of the ECM all support our hypothesis that in addition to their potential for transdifferentiating, the ACM-seeded MSCs could also generate substances that would serve as beneficial factors in the process of healing corneal wounds.
